# Perinatal and Neonatal Outcomes in Immigrants From Conflict-Zone Countries: A Systematic Review and Meta-Analysis of Observational Studies

**DOI:** 10.3389/fpubh.2022.766943

**Published:** 2022-03-11

**Authors:** Samira Behboudi-Gandevani, Razieh Bidhendi-Yarandi, Mohammad Hossein Panahi, Abbas Mardani, Christina Prinds, Mojtaba Vaismoradi

**Affiliations:** ^1^Faculty of Nursing and Health Sciences, Nord University, Bodø, Norway; ^2^Department of Biostatistics, University of Social Welfare and Rehabilitation Sciences, Tehran, Iran; ^3^Department of Epidemiology, School of Public Health and Safety, Shahid Beheshti University of Medical Sciences, Tehran, Iran; ^4^Nursing Care Research Center, Department of Medical Surgical Nursing, School of Nursing and Midwifery, Iran University of Medical Sciences, Tehran, Iran; ^5^Department of Clinical Research, University South Denmark, Odense, Denmark; ^6^Department of Midwifery Education, University College South Denmark, Esbjerg, Denmark

**Keywords:** conflict-zone countries, immigrant, neonatal outcomes, perinatal outcomes, meta-analysis

## Abstract

**Objectives:**

There are controversies regarding the risk of adverse pregnancy outcomes among immigrants from conflict-zone countries. This systematic review and meta-analysis aimed to investigate the risk of perinatal and neonatal outcomes among immigrants from conflict-zone countries compared to native-origin women in host countries.

**Methods:**

A systematic search on the databases of PubMed/MEDLINE, *Scopus*, and Web of Science was carried out to retrieve studies on perinatal and neonatal outcomes among immigrants from Somalia, Iraq, Afghanistan, Yemen, Syria, Nigeria, Sudan, Ethiopia, Eritrea, Kosovo, Ukraine, and Pakistan. Only peer-reviewed articles published in the English language were included in the data analysis and research synthesis. The odds ratio and forest plots were constructed for assessing the outcomes of interests using the DerSimonian and Laird, and the inverse variance methods. The random-effects model and the Harbord test were used to account for heterogeneity between studies and assess publication bias, respectively. Further sensitivity analysis helped with the verification of the reliability and stability of our review results.

**Results:**

The search process led to the identification of 40 eligible studies involving 215,718 pregnant women, with an immigration background from the conflict zone, and 12,806,469 women of native origin. The adverse neonatal outcomes of the risk of small for gestational age (Pooled OR = 1.8, 95% CI = 1.6, 2.1), a 5-min Apgar score <7 (Pooled OR = 1.4, 95% CI = 1.0, 2.1), stillbirth (Pooled OR = 1.9, 95% CI = 1.2, 3.0), and perinatal mortality (Pooled OR = 2, 95% CI = 1.6, 2.5) were significantly higher in the immigrant women compared to the women of native-origin. The risk of maternal outcomes, including the cesarean section (C-S) and emergency C-S, instrumental delivery, preeclampsia, and gestational diabetes was similar in both groups.

**Conclusion:**

Although the risk of some adverse maternal outcomes was comparable in the groups, the immigrant women from conflict-zone countries had a higher risk of neonatal mortality and morbidity, including SGA, a 5-min Apgar score <7, stillbirth, and perinatal mortality compared to the native-origin population. Our review results show the need for the optimization of health care and further investigation of long-term adverse pregnancy outcomes among immigrant women.

## Introduction

The number of international immigrants, particularly asylum seekers and refugees from conflict-zone countries, continues to grow rapidly. In Europe, ~1 in 10 people, is currently an international immigrant (1). According to the U.N. High Commissioner for Refugees, at the end of 2019, at least 100 million people are forcibly displaced (2). Although there is no international consensus on the definition of immigrant (1), it could be defined by the length of settlement in the host country, documentation status, voluntary or forced movement, and underlying reasons for immigration (1, 3).

Immigration has been increasingly accepted as an important determinant of health (4, 5), but the association between immigration and health status is less understood. Additionally, incorporating the mixed definition for immigration has increased the extent and complexity of this association, which can negatively impact the health status of immigrants in host countries (6). These differences are more complicated by pregnancy (7).

Female immigrants from conflict-zone countries are one of the largest groups of immigrants and have been shown to be a vulnerable population. Given that they are mainly asylum seekers and undocumented immigrants, their access to maternal healthcare services is limited, which can lead to a higher risk of mortality and morbidity, particularly among pregnant women (8–10).

There is an abundance of research on maternal and newborn health, but the results of studies on these specific populations are heterogeneous and inconclusive (11–15). Therefore, this systematic review and meta-analysis aimed to investigate the risk of perinatal and neonatal outcomes among immigrants from conflict-zone countries compared to native-origin women in host countries.

## Materials and Methods

For conducting this systematic review and meta-analysis, a review protocol was predesigned based on Cochrane's methods, which is available upon request. The review results were reported according to the Preferred Reporting Items for Systematic Reviews and Meta-Analyses (PRISMA) guideline (16). The search strategy was based on the population, intervention, comparison, outcome, and study (PICOS) design framework.

Population: pregnant women with an immigration background

Intervention: None.

Comparison: Pregnant women with a native origin background.

Outcome: Maternal and neonatal adverse outcomes.

Study design: Observational studies.

### Eligibility Criteria

Studies were eligible for inclusion if they fulfilled the following criteria: original research; international immigration from conflict-zone countries of Somalia, Iraq, Afghanistan, Yemen, Syria, Nigeria, Sudan, Ethiopia, Eritrea, Kosovo, Ukraine, and Pakistan; presenting data on at least one of the perinatal or neonatal outcomes; having the reference group from host countries, and access to the full-text. However, only peer-reviewed articles published in the English language were included in the data analysis and research synthesis. Exclusion criteria were studies without accurate and clear data on research variables, duplicated data, and studies focusing on a specific minor population such as adolescents. However, no restrictions were applied on the status or length of time for the receiving country.

### Information Sources and Search Strategy

The studies were identified by searching electronic databases, such as PubMed (including Medline), Scopus, and Web of Science (from January 1, 2000, until September 1, 2020). Also, a manual search in the references lists of selected studies and other relevant reviews was performed. The search strategy was developed with the collaboration of a librarian. Search keywords regarding immigration from conflict-zone countries and adverse pregnancy outcomes were combined and used for search (Supplementary Table 1).

### Study Selection and Extraction

Potentially relevant papers were independently screened by two reviewers (SBG and RBY). The studies were excluded when their titles and abstracts did not meet our eligibility criteria. The full text of the remaining studies was evaluated. Discrepancies were resolved by discussion between the review authors and, if necessary, by appealing to the third reviewer (MV). The following data, including the origin of the study, publication year, study period, the size of the study population, population characteristics, including age and body mass index (BMI), outcome measurements, including the number, prevalence, or risk of adverse pregnancy events, were extracted from the studies that met our inclusion criteria. To prevent bias in the data extraction and data entry, the accuracy of data before the meta-analysis was assessed through double-checking the data extraction process.

### Definition of Terms

According to the International Organization for Migration (IOM), the immigrant population has been defined as any person moving across an international border regardless of the person's legal status, whether it is voluntary or involuntary and what the causes of movement are, or how long the length of the stay is (17). In addition, conflict-zone refers to high-intensity conflict war or political instability that disrupts essential services, such as housing, transportation, communication, sanitation, water, and health care, which requires the response of people outside of the affected community. For the current study, conflict-zone counties consisted of Somalia, Iraq, Afghanistan, Yemen, Syria, Nigeria, Sudan, Ethiopia, Eritrea, Kosovo, Pakistan, and Ukraine.

### Quality Assessment for Individual Studies

Methodological structures and result presentation in the studies were assessed using the Newcastle–Ottawa scale (18). Three domains of study selection and a comparability outcome of studies were scored. Studies with quality scores above 6 were judged high quality, 4–6 moderate quality, and <4 low quality.

### Statistical Analysis and Data Synthesis

The association between immigration status and pregnancy outcomes was examined using the calculation of the pooled odds ratio (OR) (with 95% CI) as the effect size and the *p* < 0.05 were considered statistically significant.

The random-effects model described by DerSimonian and Laird was used for analysis (19). The corresponding forest plots were constructed for both the pooled prevalence and the OR of the outcomes. Study heterogeneity was assessed using the inconsistency index (I^2^-statistic), and > 50% was considered substantial heterogeneityw. In addition, sensitivity analysis was performed to show the influence of each study on the overall meta-analyses' estimates. Publication bias was assessed through the Harbord test. In case of publication bias, the trim and fill correction was applied for adjustment. Data analyses were conducted using Stata (version 14; STATA Inc., College Station, TX, USA).

## Results

**Figure 1:**
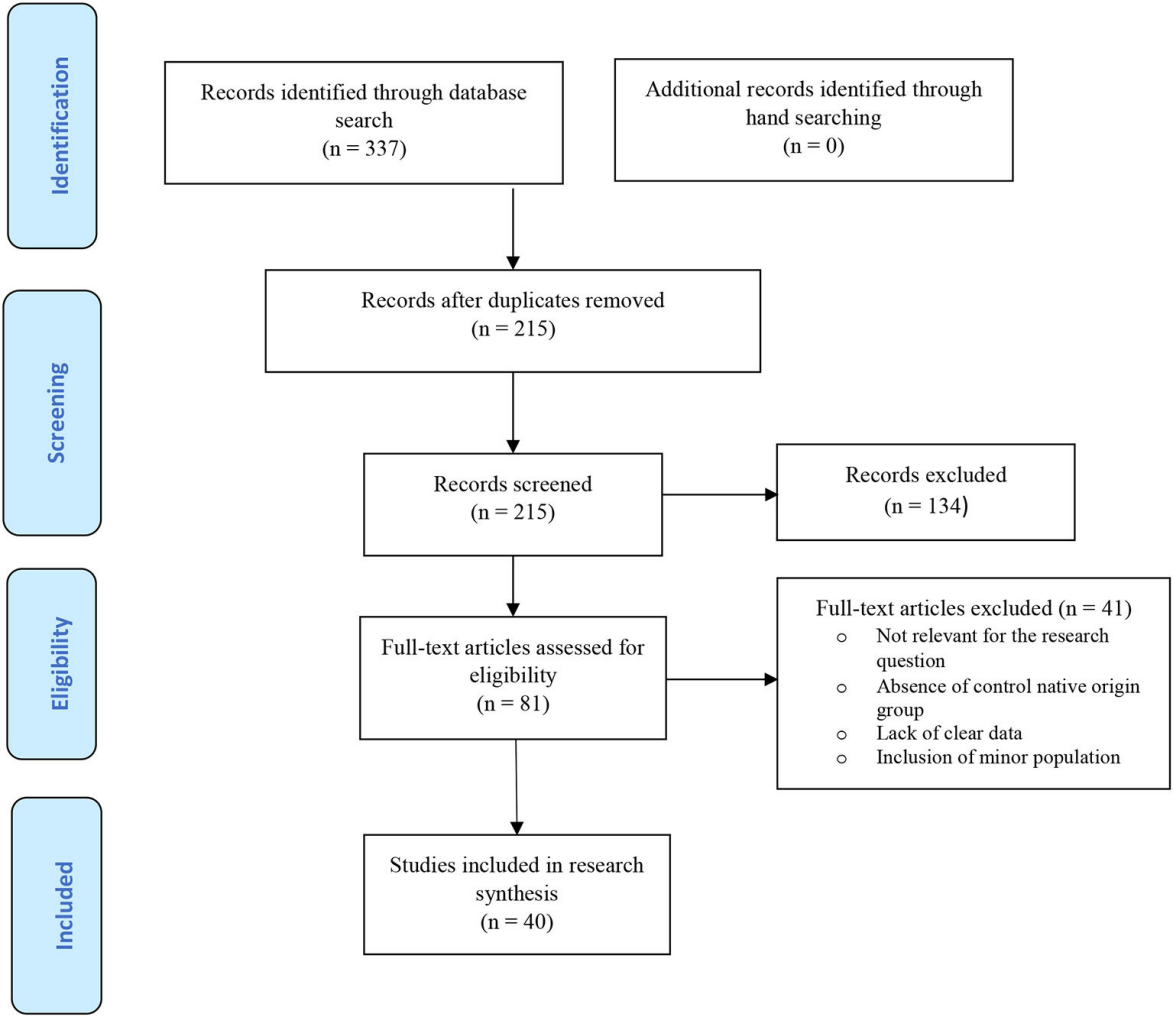
The PRISMA flowchart of the search process.

The search yielded 337 citations, including 122 duplicates (Figure 1). The screening of titles and abstracts resulted in the exclusion of 134 studies. After a full-text appraisal of 81 studies, 40 studies were included (3, 13, 20–57), involving 215,718 pregnant women with an immigration background from conflict-zone countries and 12,806,469 native-origin women. The manual search yielded no additional study. Characteristics of the included studies were summarized in Table 1. The quality appraisal of the included studies has been presented in Supplementary Tables 2, 3. A total of 30 studies were judged as high quality (3, 13, 20, 22–26, 28, 30, 32, 34, 36–38, 40–44, 46–50, 52, 54–56); 10 for moderate quality (21, 29, 31, 33, 35, 39, 45, 51, 53, 57); and no study had low quality.

**Table 1:** A baseline characteristic of the study participants.

**References**	**Data sources**	**Year of data**	**Host country**	**Native origin group sample size**	**Origin of immigrants**	**Sample size of the immigrant group**
Abdulrahim et al. (20)	Population-based data registers	2011–2013	Lebanon	45,442	Syria	4,910
Alnuaimi et al. (21)	Twogovernmentalhospitals	2014	Jordan	644	Syria	616
Badshah et al. (22)	Fourpublichospitals	2003	Pakistan	914	Afghan	125
Bakken et al. (54)	One Hospital	2006–2013	Norway	8,237	1. Pakistan, first generation	1.211
					2. Pakistan, second generation	2.76
Bakken et al. (3)	Medical birth Registry of Norway and statistic of Norway	2006–2010	Norway	6,826	1.Somalia	1.278
					2. Iraq	2.166
					3. Afghanistan	3.71
Bastola et al. (23)	Medical birth register and the hospital discharge register	2004–2014	Finland	243	Somali	584
Belihu et al. (25)	Victorian routine perinatal data registry	1999–2007	Australia	427,755	1. Eritrea	1.453
					2. Ethiopia	2.1,094
					3. Somalia	3.1,861
Belihu et al. (26)	Victorian perinatal data collection	1999–2007	Australia	203,206	1. Eritrea	1.285
					2. Ethiopia	2.695
					3. Somalia	3.1,380
Belihu et al. (26)	Victorian perinatal data collection	1999–2007	Australia	237,943	1. Eritrea	1.366
					2. Ethiopia	2.884
					3. Somalia	3.1,547
Calderon-Margalit et al. (28)	One medical	2002–2009	Israel	27,307	Ethiopia	1,319
	center					
Çelik et al. (29)	One hospital	2013–2016	Turkey	48,506	1. Syria	1.718
					2. Iraq	2.136
Col Madendag et al. (30)	One hospital database	2018–2019	Turkey	4,271	Syria	2,040
Demirci et al. (31)	One hospital	2015	Turkey	545	Syria	545
Ekéus et al. (32)	Swedish medical birth register, income and population registers	1992–2005	Sweden	1,094,146	Somalia	9,146
Erenel et al. (33)	One hospital	2013–2016	Turkey	300	Syria	300
Eskild et al. (34)	Medical birth registry of Norway, central	1999–2014	Norway	668,439	1. Somalia	1.9,281
	person registry of Norway				2. Afghanistan	2.2,113
					3. Iraq	3.7,423
Güngör et al. (35)	One hospital	2016–2017	Turkey	744	Syria	704
Johnson et al. (13)	Birth certificate data	1993–2001	1. USA, black	1.2,384	Somali	579
			2. USA, white	2.2,453		
Juárez et al. (36)	Swedish medical birth register	1999–2012	Sweden	568,684	1. Syria	1.5,528
					2. Iraq	2.20,770
					3. Iran	3.748
					4. Ethiopia	4.2,899
					5. Somalia	5.11,235
Kanmaz et al. (37)	One hospital	2013–2016	Turkey	12,198	Syria	4,802
Khanolkar et al. (38)	Medical birth registry	1982–2002	Sweden	1,435,286	1. Iraq	1.9,245
					2. Somalia	2.3,593
					3. Syria	3.3,963
					4. Ethiopia and Eritrea	4.4,364
Kiyak et al. (39)	One hospital	2016–2017	Turkey	940	Syria	616
Lubotzky-Gete et al. (40)	One hospital	1998–2011	Israel	63,405	Ethiopia	1,667
Malin et al. (41)	Finnish medical birth register, statistics Finland	1999–2001	Finland	158,469	1. Iranian, Iraqi, Afghan	1.428
					2. Somalian	2.817
Naimy et al. (42)	Medical birth registry of Norway, Norwegian central person registry	1986–2005	Norway	1,062,744	1. Somalia	1.5,410
					2. Iraq	2.4,662
					3. Afghanistan	3.3,204
Naimy et al. (43)	Medical birth registry of Norway, Norwegian central person registry	1986–2005	Norway	1,062,744	1. Iraq	1.5,410
					2. Afghanistan	2.665
Kragelund Nielsen et al. (44)	Danish medical birth registry	2004–2015	Denmark	621,154	1. Syria	1.1,768
					2. Somalia	2.5,539
					3. Afghanistan	3.3,281
					4. Iraq	4.6,150
Ozel et al. (45)	One hospital	2015	Turkey	576	Syria	576
Park et al. (46)	Live birth records provided by vital statistics	2002–2011	Canada	670,492	1. Somalia	1.4,833
					2. Afghanestan	2.6,392
					3. Ethiopia	3.2,726
					4. Iraq	4.5,406
					5. Syria	5.1,159
Pedersen et al. (47)	Danish medical birth registry	1978–2007	Denmark	1,557,944	Somali	8,555
Råssjö et al. (48)	Records of antenatal and obstetric care	2001–2009	Sweden	513	Somali	258
Sanchalika et al. (55)	Birth certificate data, hospitalization data	1999–2002	USA, white	308,508	Pakistan	2,924
Sørbye et al. (49)	Birth registry data, immigration data	1990–2009	Norway	385,306	1. Iraq	1.2,165
					2. Somalia	2.2,014
Sørbye et al. (50)	Statistic Norway, medical birth registry of Norway	1990–2009	Norway	868,832	1. Somalia	1.8,094
					2. Iraq	2.5,879
Turkay et al. (51)	One hospital	2016–2017	Turkey	7,950	Syria	620
Vangen et al. (52)	Medical birth registry of Norway	1986–1998	Norway	535,600	Pakistan	4,929
Vangen et al. (52)	Medical birth registry of Norway	1986–1998	Norway	702,192	Somalia	1,733
Yoong et al. (57)	One teaching hospital	2002	UK	61	Kosovo	61

A total of 77.5% of studies were conducted in Europe [*n* = 31, including Norway (3, 34, 42, 43, 49, 50, 52, 54, 56), Finland (23, 24), Israel (28, 40), Turkey (26–31, 33, 35, 37, 39, 45, 51), Sweden (32, 36, 38, 48), Finland (41), Denmark (44, 47), Netherlands (53), UK (57)], 7.5% in Asia [*n* = 3, including Lebanon (20), Jordan (21), Pakistan (22)], and 12.5% in others (*n* = 5, including USA (13, 56), Canada (46), and Australia (25–27)].

### Meta-Analysis of Outcomes

**Figure 2:**
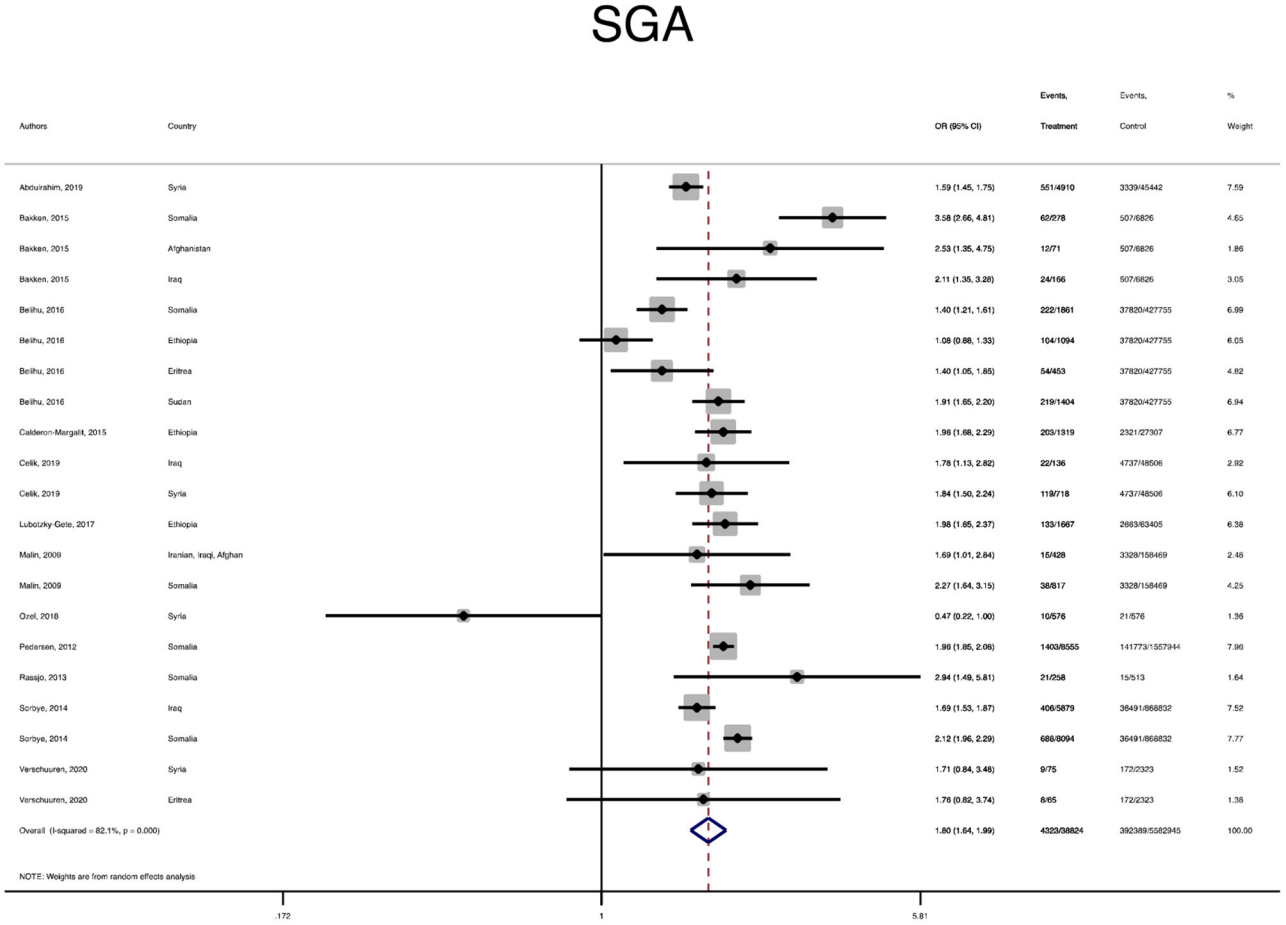
A forest plot of the pooled odds ratio of small for gestational age (SGA) in the immigrant and native-origin populations.

**Figure 3:**
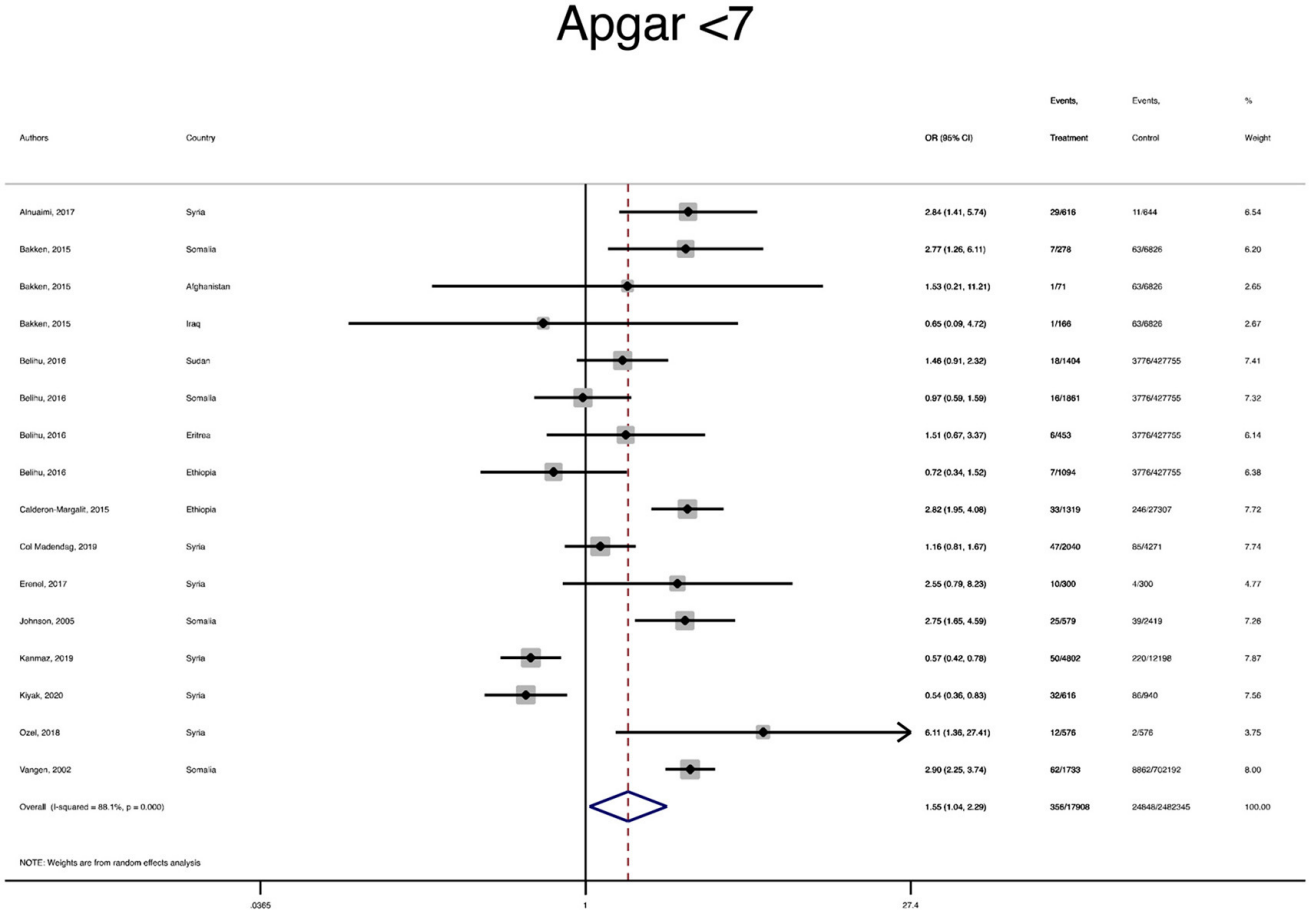
A forest plot of the pooled odds ratio of a 5-min Apgar score less than 7 in the immigrant and native-origin populations.

**Figure 4:**
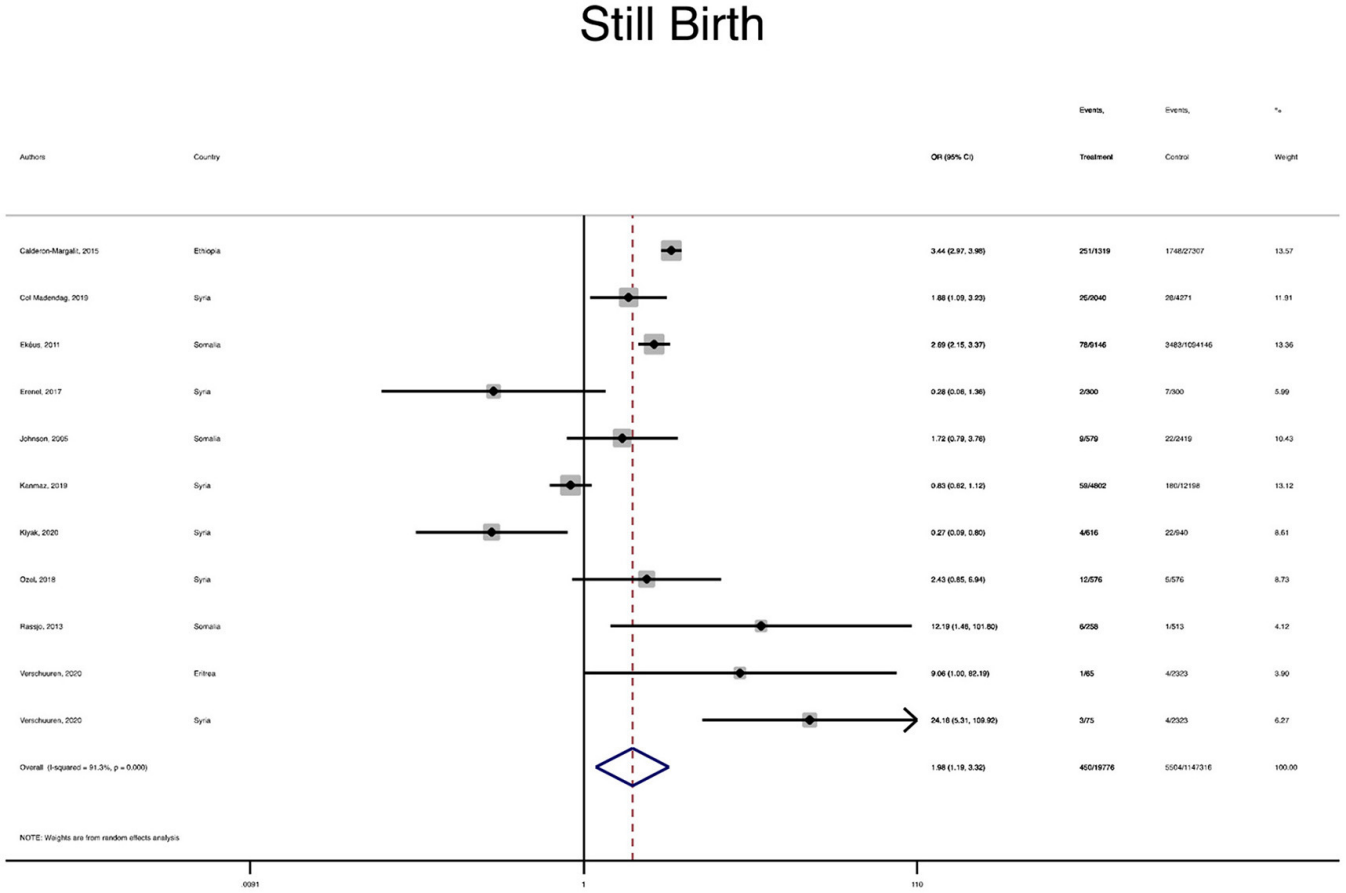
A forest plot of the pooled odds ratio of still birth in the immigrant and native-origin populations.

**Figure 5:**
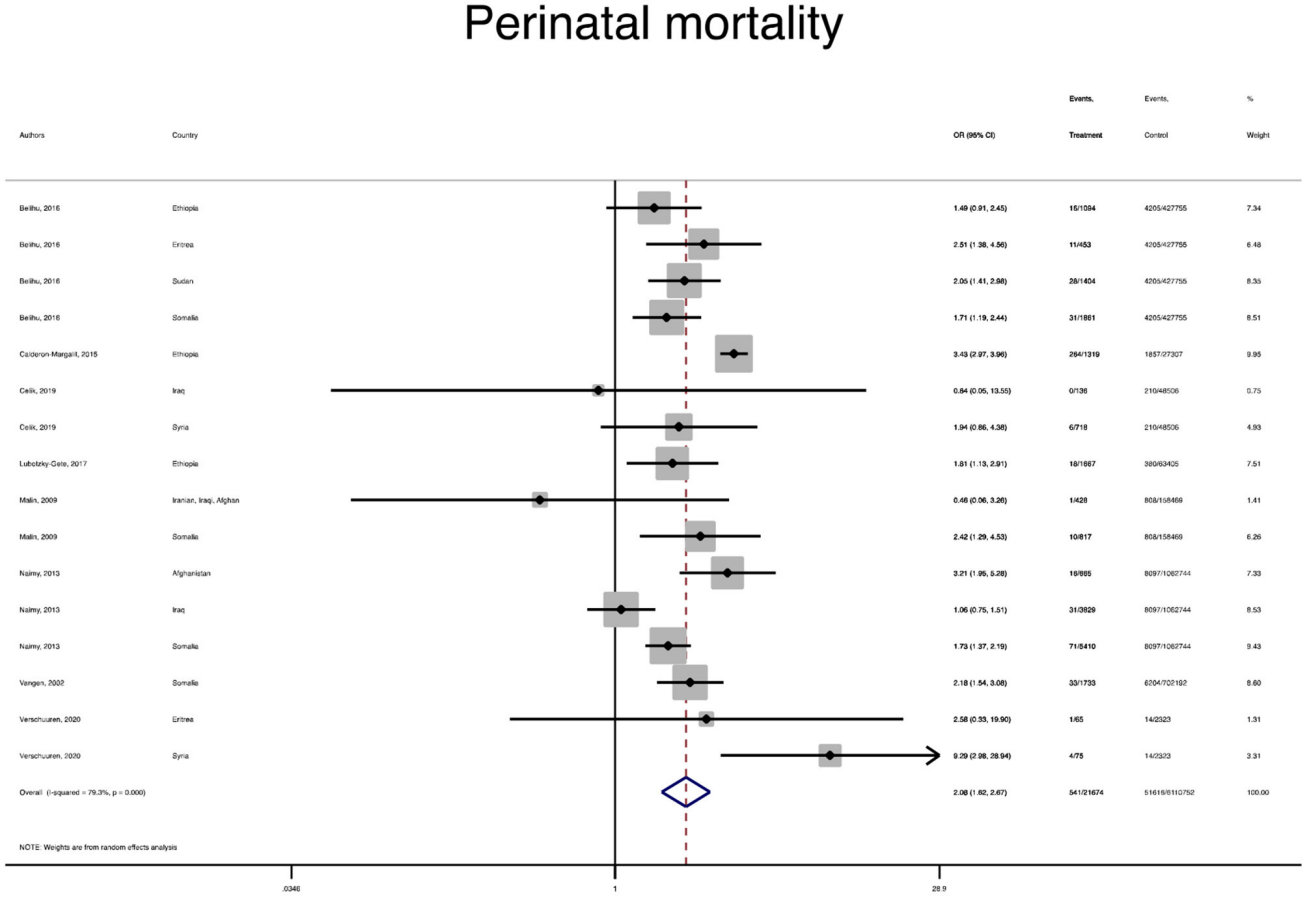
A forest plot of the pooled odds ratio of perinatal mortality in the immigrant and native-origin populations.

**Figure 6:**
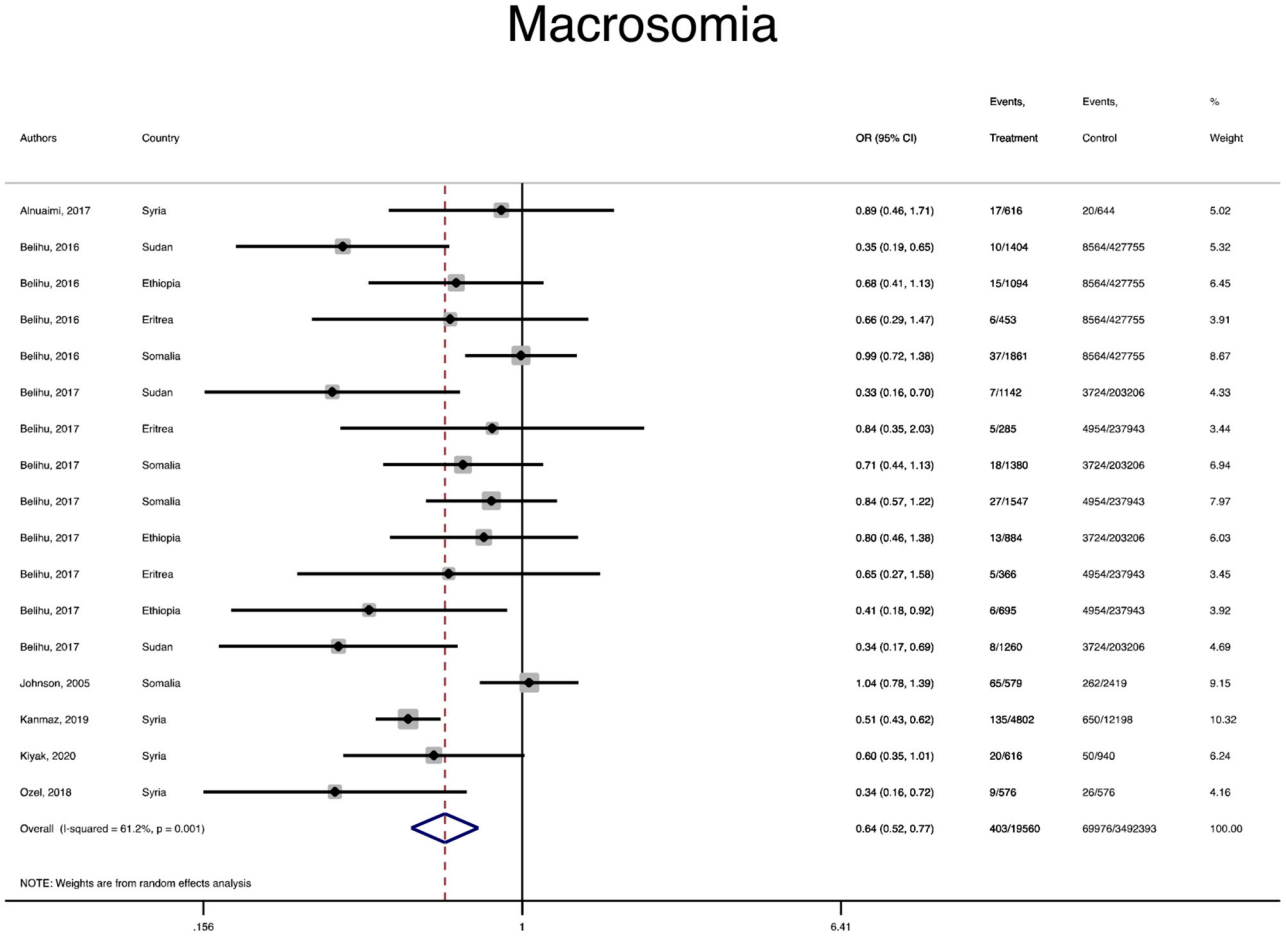
A forest plot of the pooled odds ratio of macrosomia in the immigrant and native-origin populations.

**Figure 7:**
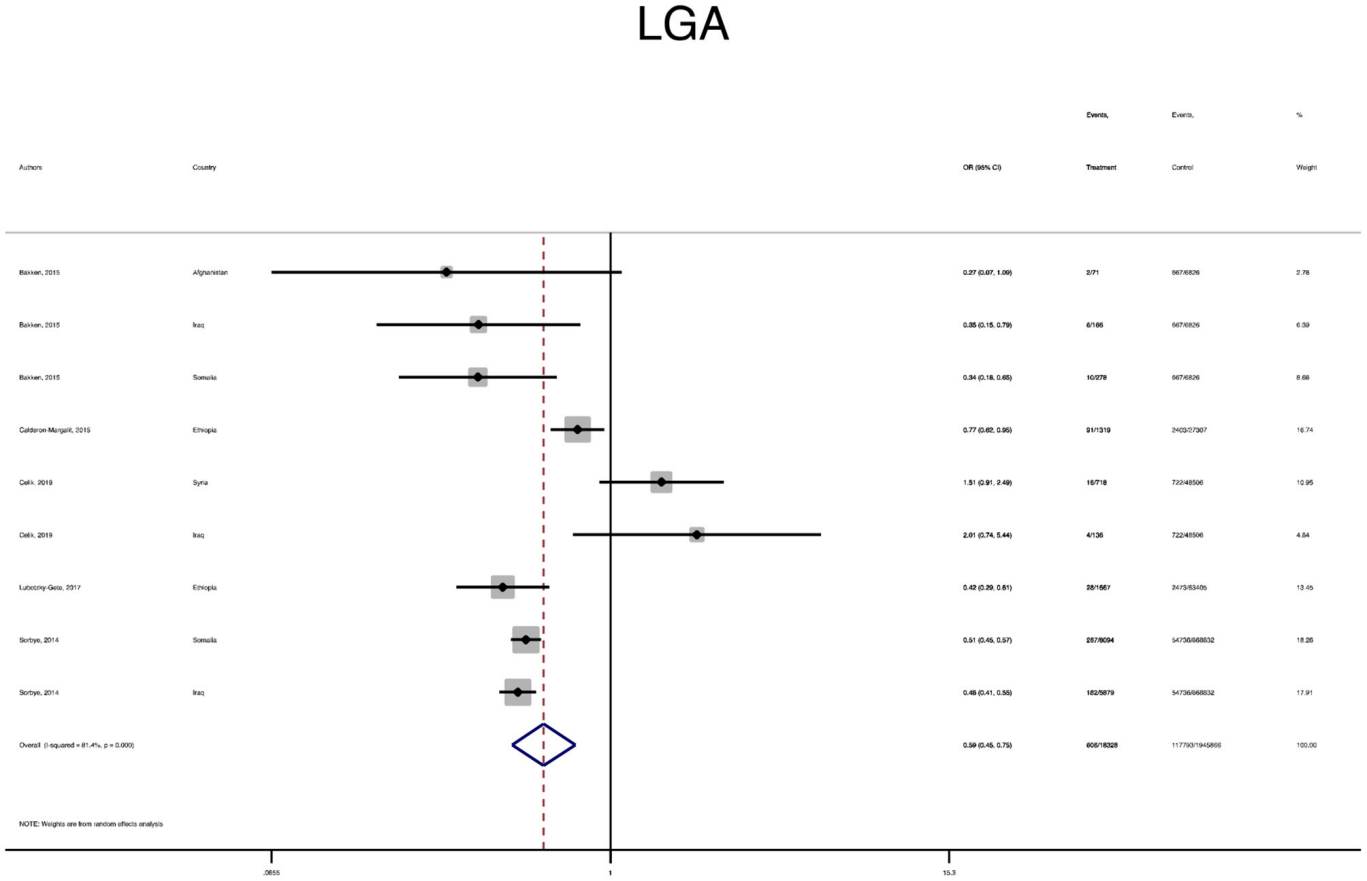
A forest plot of the pooled odds ratio of large for gestational age (LGA) in the immigrant and native-origin populations.

**Figure 8:**
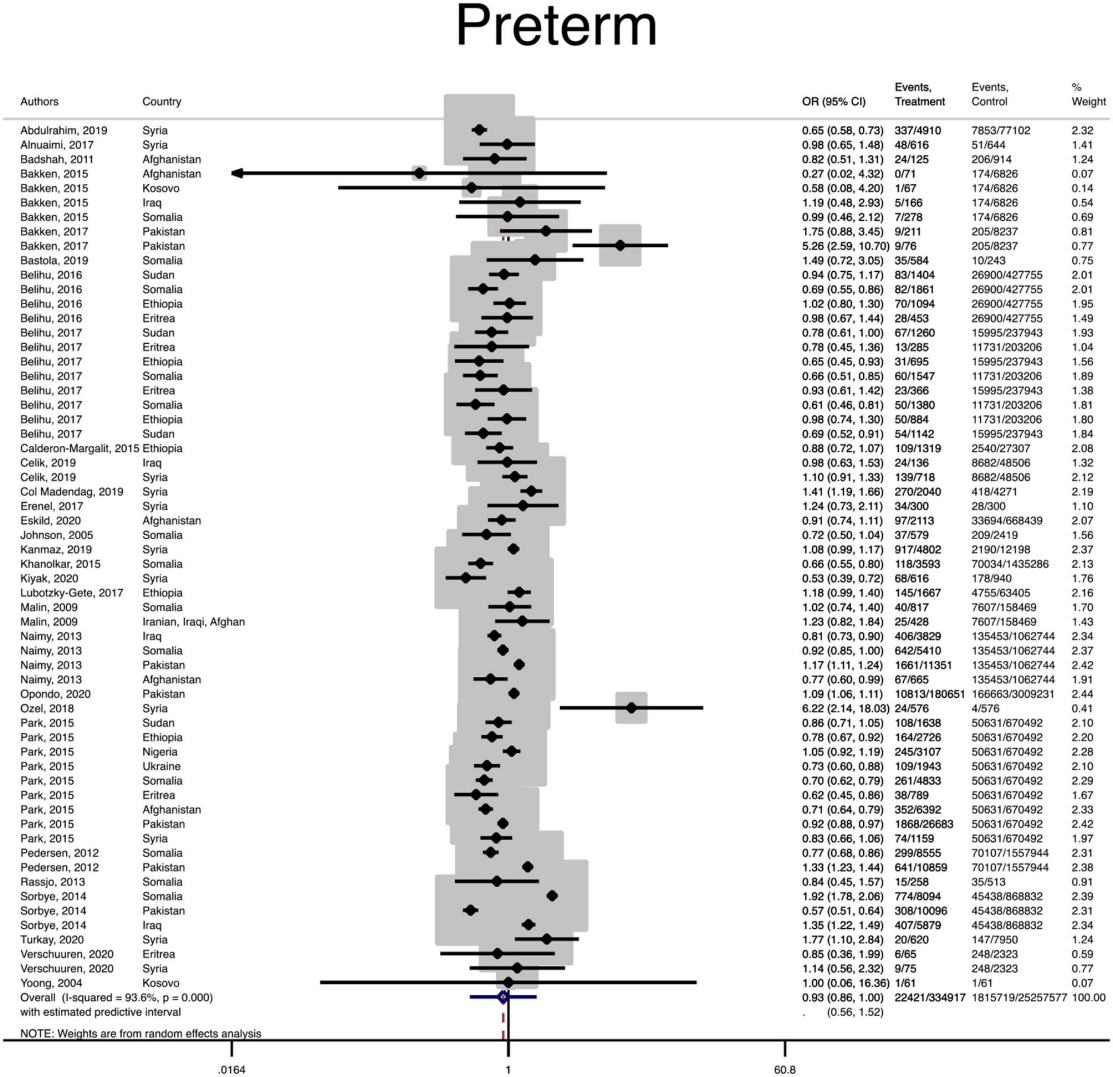
Forest plot of the pooled odds ratio of preterm birth in the immigrant and native-origin populations.

The pooled OR and estimation of heterogeneity and publication bias have been shown in Table 2. However, no substantial publication bias based on the Harbord test was observed, except for macrosomia, which was corrected using the trim and fill method of publication bias adjustment. The risk of most serious adverse neonatal outcomes, analyzed using the random-effects model in the immigrant women, was significantly higher compared to the native pregnant women. In this respect, the risk of small for gestational age (SGA) (Pooled OR = 1.8, 95% CI = 1.6, 2.1), a 5-min Apgar score <7 (Pooled OR = 1.4, 95% CI = 1.0, 2.1), stillbirth (Pooled OR = 1.9, 95% CI = 1.2, 3.0), and perinatal mortality (Pooled OR = 2.0, 95% CI = 1.6, 2.5) were all significantly higher in the immigrant women compared to the native-origin women (Figures 2–5). In contrast, the immigrant women had a lower risk of macrosomia (Pooled OR = 0.6, 95% CI = 0.5–0.7), large for gestational age (LGA) (Pooled OR = 0.5, 95% CI = 0.4–0.7), and preterm birth (Pooled OR = 0.9, 95% CI = 0.8–0.9) compared to their native counterparts (Figures 6–8). However, the risk of all maternal outcomes, including the cesarean section (C-S) and emergency C-S, instrumental delivery, preeclampsia, and gestational diabetes mellitus (GDM), were similar in both groups (Supplementary Figures 1–5).

**Table 2:** Heterogeneity, estimation of publication bias, and meta-analysis of the included studies on the prevalence of adverse maternal and neonatal outcomes.

**Outcome**	Publication bias Harbord test^*^	**Heterogeneity I-squared**	**Pooled OR (95%CI)**
**Adverse perinatal outcome**			
Cesarean section	0.464	98.2%	0.9 (0.8, 1.0)
Emergency cesarean section	0.091	84.7%	1.2 (0.8, 1.8)
Labor induction	0.812	94.4%	0.8 (0.7, 1.0)
Instrumental delivery	0.456	80.3%	0.9 (0.7, 1.1)
Preeclampsia	0.457	95.2%	0.8 (0.6, 1.2)
GDM	0.614	**95.5%**	1.5 (1.0, 2.0)
**Adverse neonatal outcome**			
Macrosomia	0.145	61.2%	**0.6 (0.5, 0.7)**
LGA	0.368	81.4%	**0.5 (0.4, 0.7)**
SGA	0.798	82.1%	**1.8 (1.6, 2.1)**
5-min Apgar score less than 7	0.121	88.1%	**1.4 (1.0, 2.1)**
Preterm birth	0.181	92.6%	**0.9 (0.8, 0.9)**
Still birth	0.907	91.3%	**1.9 (1.2, 3.0)**
Perinatal mortality	0.524	79.3%	**2.0 (1.6, 2.5)**

*GDM, gestational diabetes; LGA, large for gestational age; SGA, small for gestational age; NICU, neonatal intensive care unit; RDS, respiratory distress syndrome*.

*Bold values indicate statistical significance*.

* *Obtained from the trim and fill method of publication bias adjustment*.

### Sensitivity Analysis

The results were highly consistent with the main results of data analysis, and no substantial modification of the estimates of change was reported after the exclusion of individual studies (Supplementary Figures 6A–M).

## Discussion

This systematic review and meta-analysis showed that, among the immigrants from conflict-zone countries, the risk of adverse neonatal outcomes, including SGA, a 5-min Apgar score <7, stillbirth, and perinatal mortality, was higher compared with the native-origin women. However, the risk of adverse maternal outcomes such as C-S and emergency C-S, labor induction, instrumental delivery, preeclampsia, and GDM was similar in both groups.

There is no consensus on the definition of an immigrant in the international literature. Immigration has a significant impact on public health outcomes (58). It is well-documented that diversity in genetics, environment, behavior, and, also, the disparity in the socioeconomics situation at both individual and societal levels can influence the health status of immigrants (6). However, the health effects of armed conflicts have remained unexplored (59). Generally, some studies have reported similar pre-migration exposures to armed conflicts and post-migration health problems among immigrants, which are both generally poorer than the population of host counties (60, 61). However, it is argued that pregnancy may increase the risk of health problems among immigrants. Our review revealed that the risk of maternal outcomes was comparable, but the risk of most serious adverse neonatal outcomes among the immigrant women from conflict-zone countries was significantly higher than those women in host countries. In this respect, being an immigrant from a conflict-zone country does not guarantee that he or she has been exposed to trauma or violence. Nevertheless, he or she has greater exposure to perceived threats of violence, chronic stress, and physical violence (62, 63), which may have led to health problems (64).

Female refugees of child-bearing age are more vulnerable to stress exposure, which can increase the risk of preventable adverse neonatal outcomes. Moreover, many of these adverse neonatal outcomes are related to preventable risk factors such as difficulties in access to timely prenatal care in host countries (65). However, due to multiple underlying reasons such as language and cultural barriers, and the policy and social systems in host countries, these women tend to initiate prenatal care lately and have fewer prenatal visits (66). It should be noted that many of these adverse outcomes not only have immediate adverse effects on neonates but also can have long-lasting consequences on the growth and development of the newborn well into adulthood (66, 67).

We found that the risk of adverse maternal outcomes among the immigrants was similar to the native population. Some studies have found a “healthy-migrant effect” among immigrants, which is a similar or a better health status than the population in host countries (68). It is hypothesized that immigrants represent a selectively healthy and young group of people, and, therefore, their health status stands out compared to the general native population in host countries (69–71). The effect of the healthy immigrants may be translated across some maternal outcomes among immigrants' mothers from conflict-zone countries. In addition, Bakken et al. (3) argued that women originating from various conflict-zone countries may experience different risks of adverse perinatal outcomes. They reported that immigrant women from Somalia needed more targeted care during pregnancy and childbirth than those from Kozovo (3).

Despite our review results that exclusively focus on pregnant women from conflict-zone countries, our meta-analysis summarized available evidence regarding the perinatal health issues among these groups of women. The quality of the included studies was either moderate or high. Since no study was considered to be of poor quality, it helped us to represent the acceptable quality of evidence in this meta-analysis.

The main limitation of this study was the lack of data on potential risk factors in included studies, which might have potentially affected the outcomes of interest. In addition, the rate of iatrogenic C-S due to GDM and pre-eclampsia was not reported due to the lack of data in the included original manuscripts. Although many war immigrants are undocumented, the lack of data on the maternal duration of residence in host countries, as well as heterogeneity between immigrants, asylum seekers, and refugees did not let us perform subgroup analysis among them.

## Conclusion

In conclusion, our systematic review and meta-analysis demonstrated that the risk of some adverse maternal outcomes was comparable, but the immigrant women from conflict-zone countries had a higher risk of neonatal mortality and morbidity, including SGA, a 5-min Apgar score <7, stillbirth, and perinatal mortality compared to the native-origin population. This study shows the need for the application of preventive strategies to prevent missing valuable opportunities and to optimize health across two generations. Further investigation into long-term adverse pregnancy outcomes for this population is warranted.

## Data Availability Statement

The original contributions presented in the study are included in the article/Supplementary Material, further inquiries can be directed to the corresponding author/s.

## Author Contributions

All authors listed have made a substantial, direct, and intellectual contribution to the work and approved it for publication.

## Funding

Nord University, Bodø, Norway covered the processing charge to this article.

## Conflict of Interest

The authors declare that the research was conducted in the absence of any commercial or financial relationships that could be construed as a potential conflict of interest.

## Publisher's Note

All claims expressed in this article are solely those of the authors and do not necessarily represent those of their affiliated organizations, or those of the publisher, the editors and the reviewers. Any product that may be evaluated in this article, or claim that may be made by its manufacturer, is not guaranteed or endorsed by the publisher.
