# Supplementary material for: Perinatal and Neonatal Outcomes in Immigrants From Conflict-Zone Countries: A Systematic Review and Meta-Analysis of Observational Studies
Source: Front Public Health. 2022 Mar 11;10:766943. doi: 10.3389/fpubh.2022.766943 (PMC8962623; doi:10.3389/fpubh.2022.766943)
Supplement: Supplementary file 1 [file Data_Sheet_1.docx]

**Supplementary file**

Supplementary table 1. Key words used for search in this review.

| (immigration OR migration OR immigrant OR migrant OR emigrant OR asylum seeker OR asylum seeking OR asylum OR refugee) AND (Somalia OR somalian OR Iraq OR Afghanistan OR afghan OR Yemen OR Syria OR Syrian OR Nigeria OR Sudan OR Ethiopia OR Eritrea) AND (“adverse pregnancy outcomes” OR “pregnancy outcomes” OR “pregnancy complications” OR “abortion” OR “miscarriage “OR “pregnancy loss” OR “fetal death” OR “stillbirth” OR “preeclampsia” OR “gestational hypertension” OR PIH OR “gestational diabetes” OR “hemorrhage” OR “postpartum hemorrhage” OR PPH OR “Placenta abruption” OR “placenta previa” OR “preterm” OR “premature rupture of membrane” OR PROM OR “Intra uterine growth restriction” OR IUGR OR “Low birth weight” OR LBW OR “oligohydramnios” OR Apgar OR “fetal distress” OR “neonatal distress” OR RDS OR “neonatal death” OR “neonatal mortality” OR “neonatal admission” OR “NICU admission” OR “malformation” OR “anomalies” OR “birth weight” OR LGA OR “large for gestational age” OR SGA OR “small for gestational age” OR “gestational diabetes” OR GDM OR IUFD OR “intra uterine fetal death” OR cesarean OR “operative delivery” OR “instrumental delivery” OR vacuum) |
| --- |

Supplementary table 2: Quality assessment of the included studies using the Newcastle–Ottawa Quality Assessment Scale for cohort studies.

|  | **SELECTION** | | | | **COMPARABILITY** | **Outcome** | | | **Total scores** | **Study Quality** |
| --- | --- | --- | --- | --- | --- | --- | --- | --- | --- | --- |
| **Author, Year** | Representativeness of the exposed cohort * | Selection of the non-exposed cohort * | Ascertainment of exposure * | No outcome of interest at the start of study * | A: Study controls for age and/or BMI *  B: Study controls for *other confounders | A: doctor’s diagnosis OR objective measurements  B: parent/self-reported doctor’s diagnosis OR use of medication | Follow-up long enough for outcomes * | Adequacy of follow up of cohorts * |  |  |
| Alnuaimi et al, 2017 | - | - | * | * |  | * | * | * | 5* | Moderate |
| Badshah et al, 2011 | - | - | * | * | ** | * | * | * | 7* | High |
| Bakken, 2015 | * | * | * | * | ** | * | * | * | 9* | High |
| ÇELIK et al, 2019 | - | - | * | * | - | - | * | - | 3* | Low |
| Yoong et al, 2004 |  |  | * | * |  | * | * | * | 5* | Moderate |
|  |  |  |  |  |  |  |  |  |  |  |
|  |  |  |  |  |  |  |  |  |  |  |
|  |  |  |  |  |  |  |  |  |  |  |
|  |  |  |  |  |  |  |  |  |  |  |
|  |  |  |  |  |  |  |  |  |  |  |
|  |  |  |  |  |  |  |  |  |  |  |
|  |  |  |  |  |  |  |  |  |  |  |
|  |  |  |  |  |  |  |  |  |  |  |
|  |  |  |  |  |  |  |  |  |  |  |
|  |  |  |  |  |  |  |  |  |  |  |
|  |  |  |  |  |  |  |  |  |  |  |

Supplementary table 3. Quality assessment of the included studies using the Newcastle–Ottawa Quality Assessment Scale for cross-sectional study.

|  | **SELECTION** | | | | **COMPARABILITY** | **Outcome** | | **Total scores** | **Study Quality** |
| --- | --- | --- | --- | --- | --- | --- | --- | --- | --- |
| **Author** | Representativeness of the samples | Sample size | Non-responders | Ascertainment of the exposure | A: study controls for age and/or BMI  B: control for any additional factor | Assessment of the outcome  a) Independent blind assessment.  b) Record linkage.  c) Self report. | Statistical test |  |  |
| Abdulrahim et al. 2018 | * | * | * | * | ** | ** | * | 9* | High |
| Bakken et al, 2017 |  | * |  | * | ** | ** | * | 7* | High |
| Bastola et al, 2019 | * | * | * | * | ** | ** | * | 9* | High |
| Bastola et al, 2020 | * | * | * | * | ** | ** | * | 9* | High |
| Belihu et al, 2016 | * | * | * | * | ** | ** | * | 9* | High |
| Belihu et al, 2017 | * | * | * | * | ** | ** | * | 9* | High |
| Belihu et al, 2017 | * | * | * | * | ** | ** | * | 9* | High |
| Calderon-Margalit et al, 2015 |  | * | * | * | ** | ** | * | 7* | High |
| Erenel et al, 2017 |  |  |  | * | ** | ** | * | 6* | Moderate |
| Eskild et al, 2020 | * | * | * | * | ** | ** | * | 9* | High |
| Juarez et al, 2017 | * | * | * | * | ** | ** | * | 9* | High |
| Khanolkar et al, 2015 | * | * | * | * | ** | ** | * | 9* | High |
| Malin and Gissler, 2009 | * | * | * | * | ** | ** | * | 9* | High |
| Naimy et al, 2013 | * | * | * | * | ** | ** | * | 9* | High |
| Naimy et al, 2015 | * | * | * | * | ** | ** | * | 9* | High |
| Park et al, 2015 | * | * | * | * | ** | ** | * | 9* | High |
| Pedersen et al, 2012 | * | * | * | * | ** | ** | * | 9* | High |
| Sanchalika and Teresa, 2015 | * | * | * | * | ** | ** | * | 9* | High |
| Sørbye et al, 2015 | * | * | * | * | ** | ** | * | 9* | High |
| Sørbye et al, 2014 | * | * | * | * | ** | ** | * | 9* | High |
| Vangen et al, 2000 | * | * | * | * | ** | ** | * | 9* | High |
| Vangen et al, 2002 | * | * | * | * | ** | ** | * | 9* | High |
| Verschuuren et al, 2020 |  |  | * | * |  | ** | * | 5* | Moderate |

Supplementary figure 1. Forest plot of the pooled odds ratio of cesarean section in the immigrant and native origin populations.

Supplementary figure 2. Forest plot of the pooled odds ratio of emergency cesarean section in the immigrant and native origin populations.

Supplementary figure 3. Forest plot of the pooled odds ratio of instrumental delivery in the immigrant and native origin population.

Supplementary figure 4. Forest plot of the pooled odds ratio of preeclampsia in the immigrant and native origin populations.

Supplementary figure 5. Forest plot of the pooled odds ratio of gestational diabetes in the immigrant and native origin populations.

Supplementary figure 6. Plots of sensitivity analysis of results for (A) Macrosomia, (B) Large for gestational age, (C) Small for gestational age, (D) Apgar <7, (E) Labor induction, (F) Instrumental delivery, (G) Cesarean section, (H) Emergency caesarean, (I) Preeclampsia, (J) Preterm birth, (K) Still birth, (L) Perinatal mortality, (M) Gestational diabetes

| **A: Macrosomia** |
| --- |
| **** |
| **B: Large for gestational age** |
| **** |

| **C: Small for gestational age** |
| --- |
| **** |
| **D: Apgar <7** |
| **** |

| **E: Labor induction** |
| --- |
|  |
| **F: Instrumental delivery** |
|  |

| **G: Cesarean section** |
| --- |
|  |
| **H: Emergency caesarean** |
|  |

| **I: Preeclampsia** |
| --- |
| **** |
| **J: Preterm birth** |
| **** |
| **K:Still birth** |
| **** |
| **L: Perinatal mortality** |
| **** |
| **M: Gestational diabetes** |
| **** |
